# Effectiveness of a small breast screening programme: 25 year evaluation (25 year breast screening evaluation)

**DOI:** 10.1259/bjro.20180018

**Published:** 2019-07-13

**Authors:** Andrew Patric Nisbet, Andrew Borthwick-Clarke, Nic Scott, Helen Goulding, Harwood Jane

**Affiliations:** 1 Jersey General Hospital,

## Abstract

**Objective:**

To evaluate mammography screening quality on the Island of Jersey over a 25-year period from Jan 1990 to end March 2015 from females invited between ages 50 to 75 using a 2 yearly screening interval. Jersey had a population of only around 67,000 at onset, rising to around 100,000 at the end of the 25 years.

**Methods:**

An analysis was performed of key routinely collected measures that are important to determining if a screening programme is on course to reduce breast cancer mortality such as uptake, recall rates, screen detected cancer and interval cancer rates. Further supporting indicators including grade, stage and comparative deaths from breast cancer in screen detected and not screen detected females were also assessed.

**Results:**

Over the 25-year period 19,768 females were invited to screening and 16,866 attended, giving an uptake of 85.2%. There were 501 screen detected cancers of which 400 were invasive, and 101 DCIS. 125 interval cancers presented outside screening over the 25 years. The annual recall rate over the last 20 years was <6% for prevalent round and 4% for incident round screening. Based on the standardized detection ratio (SDR) and uptake, the estimated reduction in mortality from breast cancer was calculated as 40.2%.

**Conclusions:**

Recommended population sizes for breast units range from a quarter to half a million people. For very small units like Jersey serving smaller populations, rigorous quality control is essential to maintain credibility. Despite the small size of the programme evidence shows a similar detection rate to the UK NHS Breast screening programme was achieved. In small programmes careful monitoring of rates of uptake, recall, cancer detection and interval rates are required over adequate time periods together with supporting information to show that small units can achieve national standards and detection rates necessary to reduce breast cancer mortality.

**Advances in knowledge:**

Running a small breast cancer screening programme is challenging for quality control. The impact on mortality can be predicted for small screening programmes despite their size. 10-year group survival in screen detected invasive breast cancer >90%. Interval cancers are more advanced than screen detected invasive cancers, so high suspicion is still required in breast symptoms after "normal" screen result. Mortality in lapsed/ceased attenders suggest that extending age range could be beneficial.

## Introduction

In the 1980’s, breast cancer was largely managed by general surgeons without specialist interest. Minimal or no imaging preoperatively was commonplace. Whilst early models of mammography and xero-mammography were available improvements rapidly occurred in film/screen, processing and uniform breast compression as breast screening programmes expanded. No quality assurance (QA) was established at this time. The Forrest Report in 1986 recommended the development of a National Breast Screening Programme across the United Kingdom with quality assurance built in from the start.

The following decade encompassed a significant improvement in care with the development of specialist surgeons, breast imaging radiologists, pathologists, nurses and oncologists/radiotherapists plus the National Quality Assurance Programme and Multidisciplinary Team Management.

A National Programme for Breast Screening (NHSBSP) was introduced in the UK in 1988 following the recommendations of the Forrest Report. The Breast Screening service was established in Jersey in 1990. Initial results of the programme in Jersey were published in 1999^1^ ; these showed a significant reduction in diameter of invasive breast cancer in screened females aged 50–65 (all cancers—screen detected, interval and late cancers in ceased attenders) when compared to local females, unscreened, presenting at a rapid access diagnostic centre.

Breast screening by mammography remains controversial. The randomized trials have generated more reviews of the trials than the trials themselves.^2^ In the face of continued academic criticism, it is clear that mammographic screening must be performed in an optimal fashion, with integral quality control. The Canadian National Breast Screening Study 25 year follow up, Miller et al,^3^ has shown that a programme without modern mammographic quality control is ineffective. In Europe, mammographic screening programmes in 25 countries show a wide variation between programmes in numbers of examinations per screening unit; with implications for low volume units requiring additional efforts and the need for maintaining high level of quality across a large number of screening units for national programmes.^4^

Jersey is a small island off England’s south coast, with a programme enabling the minimum number of mammograms, read per individual film reader, recommended in the UK (5000/year). The programme screened 1833 females in the first year, 1990, rising to 2189 in 1995; 3535 in 2000; 4050 in 2005; 4464 in 2010; and 5377 in 2015. This increase reflects a rapidly increasing island population, (65,000 increasing to 100,000+), and acceptance of breast screening by females, plus the effect of repeat (incident) round screening and age extension. Also, symptomatic mammogram and double reading, enabled the individual screening radiologists to achieve the recommended target of 5000. The Screening Programme has run for over 25 years, with quality control embedded since inception, using the Quality Assurance protocols of the NHS Breast Screening Programme, with external assessment performed annually and in more detail every three years. The results detailed below in the study of Jersey females includes the decade of change plus the later years of improved care and potentially better outcomes.

The study objectives were to show that QA is possible in small programmes. We feel that QA is vital, especially in small breast screening programmes. Some information is directly interpretable, the other information is supportive rather than definitive. The most directly relevant are recall rates and cancer detection rates which can be used to monitor performance. Other information such as breast cancer deaths can be useful but only as supporting information. The study is a retrospective evaluation to collate information to help guide our (and potentially other) small programmes. The mortality and net group survival results were intended to support the aim of reducing breast cancer mortality, rather than to prove them as randomized controlled trials would be required for proof.

## Methodology

When the Jersey Breast Screening Programme commenced females aged 50–65 were invited to participate in the free programme. The lack of a population database made it difficult to invite all eligible females; the coverage has increased as the programme has progressed. Screening commenced, and continued, with prevalent round two view mammography [mediolateral oblique (MLO) and craniocaudal (CC) views] using new mammography and film processing equipment. Positioning, exposure factors and film processing followed the two counties (Falun, Sweden) trial protocols. Extended processing and an optical density of >1.4 were in place from the start. Training was obtained from UK and Swedish training centres. Local radiographers were trained primarily by technicians from the screening unit in Falun. The radiologist training was undertaken in the UK and received from Professor Tabar in Sweden^5^ and elsewhere.

In 1990, the screening interval was 3 years, with single view (MLO) at incident screen. At that time only fine needle aspiration for cytology was performed. Soon after 18g core biopsies commenced, progressing to 16g and then 14g. By the late 1990s, 11g vacuum assisted core biopsy had been introduced. In 1995, the screening interval was reduced to 2 yearly, with two view incident round screening and the upper age limit was raised to 69. From 2000, females aged 69–75 were allowed to self-refer for 2-yearly screening. Double reading with consensus has been in place since commencement of the programme. Females recalled for assessment after screening were examined by further mammographic imaging (spot compression or magnification views). Ultrasound of the breast was used in assessment as was fine needle aspiration from commencement. Core biopsy and vacuum assisted biopsies were introduced as these became available.

Initially general surgeons and non-specialist trained pathologists were involved. Breast specialist histopathology became available in 1994 and dedicated breast surgeon care commenced and gradually took over from 1997 onwards. Tamoxifen use increased in the late 1990s and Oncology/Radiotherapy provision improved from 1999. Sentinel node biopsy was instigated in 2001.

The service is assessed according to the NHSBSP QA standards (Supplementary material 1).^6^ External review by UKBSP QA assessors is performed in administration, radiographic and reporting (radiologist performance) components of the service with annual review of the results.

Supplemental Material

The study reviewed the results of 25 years, concentrating on the essential uptake rate, recall rate and cancer detection. Supporting documentation of grade, staging, interval cancer rate and mortality distribution were included to detect any potential points of failure in the programme.

### Objectives

1. Evaluate the quality and early indication of effectiveness; based on compliance with NHSBSP QA guidelines, external reviews and size, grade and node status of screen detected invasive cancers.
2. Classify deaths from invasive breast cancer in females invited for screening in the 25 years, according to presentation (*e.g.* invited but never attended, cancers in lapsed/ceased attenders, interval cancer and screen detected cancer).
3. Describe the percentage of females surviving for at least 10 years after primary breast cancer screen detected diagnosis, corrected for deaths from other causes and females lost from the programme (This is an indicative performance measure).

### Data analysis

The study size included all females invited/screened over the 25-year period. Invasive cancers were grouped together rather than analysed separately (*e.g.* ductal, lobular etc.) as were DCIS, owing to the small total numbers. Similarly, mortality statistics were grouped together rather than based on tumour type, grade, stage or number of times screened before presentation, again owing to the small total numbers. Missing data was addressed by searching hospital records—both computer held and old paper files, plus letters to general practitioners, managers, and doctors, to obtain current information of status and address. If pathology reports were missing, or the histology had been reported by a non-breast specialist histopathologist, the sections were reviewed and a new report was obtained by a specialist breast histopathologist.

TMN staging was by UICC TNM classification. Females who had had multiple screens were included as separate data if they had a new primary cancer detected on separate screening episodes, after they had been discharged from breast clinic follow up. The new primary screen detected cancer was classified by the breast multidisciplinary team, as a new lesion. Five females were in this category.

The diameter of tumour and axillary node status was only used if available from an experienced breast pathologist report. Pathology reports were individually reviewed retrospectively for all screen detected and interval cancers. Diameter, grade and node status were only included in analysis if a known breast trained histopathologist had reviewed it. Interval cancer was identified at MDT meetings and from annual public health documentation. Interval cancers were reviewed annually, using data supplied to the breast multidisciplinary team and the public health department.

Vital Status was assessed by Public Health Department Mortality results from breast cancer (death certificates) and proof of life from either recent attendance for imaging or pathology sample, or confirmation from patients’ GP.

Comparison between risks of breast cancer deaths in those attending screening or never attending and the comparison between Stage II + screen detected and interval invasive cancers was by the test of two proportions (prtesti command in STATA).

Recall rate was based on past 20 years to reflect the stable period of regular 2 yearly, two view mammography. The estimated breast cancer mortality reduction was based on relative uptake and age standardized invasive cancer detection rate using the mortality reduction achieved by the Swedish-Two County randomized controlled trial^5^ and calculated using (uptake/90) × SDR × 31.

The SDR was introduced by Dr. R Blanks et al. in 1996^7^ to the UK breast screening programme to standardize assessment. The SDR can be corrected for age and background incidence based on geographical location. The SDR is in routine use in the UK and has been applied to programmes in Europe and Australia.

## Results

### Objective 1

The evaluation of the quality and early indication of effectiveness based on compliance with NHS BSP QA guidelines, external reviews and size, grade and node status of screen detected invasive cancers. The number of females invited, screened and cancers detected over 25 years is shown in Table 1.

**Table 1.** Number of females invited, screened and cancers detected over the 25 years

	Jersey results	NHSBSP achievable standard (revised 2017)
Total invited	19,768	
Total screened	16,866 (85.3%)	
Recall rate (past 20 years) Prevalent round Incident round	<6% <4%	<7% <5%
Total number of females recalled (Approximately 1:7 chance of a female being recalled over 12 screening rounds).	2,558 (15.2%)	
Benign surgical biopsies	121 (0.7%)	
Invasive cancers detected by screening	400	
DCIS detected by screening	101	
Interval cancers	125	
SDR over 25 years: minimum SDR target >1.0	1.37	≥1.4
Approximate figures for numbers of rescreens:		
Single screening episode	14%	
2–5 screening episodes	54%	
Greater than five screening episodes	32%	

SDR, standardized detection ratio.

Note 1: The Standardised Detection Rate (a ratio of the observed number of screen detected cancers divided by the expected number, based on the Swedish two county study)^ 5^.

Note 2: Recall rate includes technical recalls.

Note 3: The percentages of females recalled, biopsied etc. is based on number of females screened rather than invited.

Whilst uptake is high at 85.3% and can be accurately measured, most other measures suffer from considerable statistical instability from year to year.

**Figure 1.**
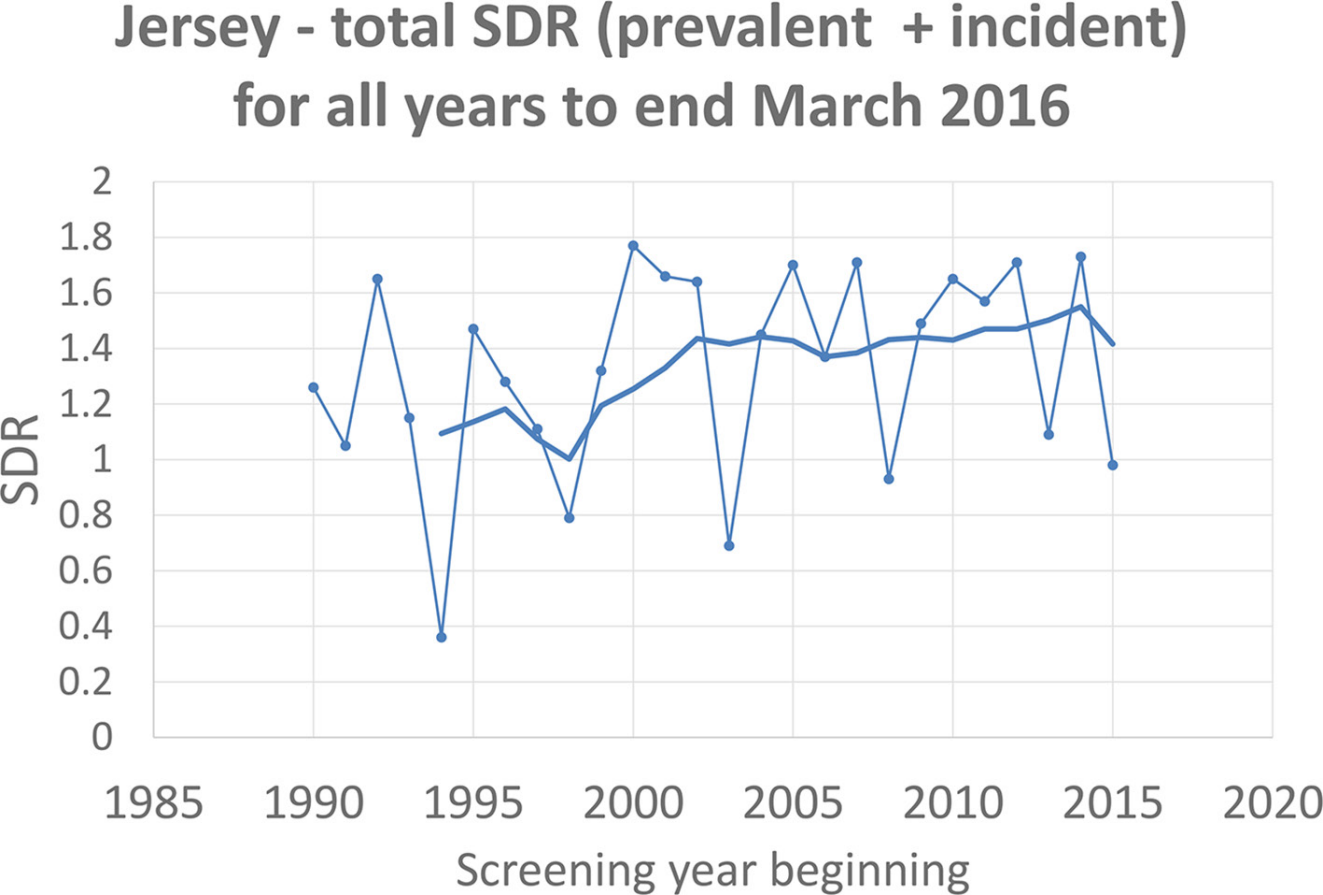
Invasive cancer detection rate measured by annual SDR with 5 year moving average. SDR,standardized detection ratio.

The mean age of females with screen detected invasive cancer was 59.2 years with a mean diameter (measured on 374 invasive cancers) of 15.6 mm. Figure 1 shows the SDR (a ratio of the observed number of screen detected cancers divided by the expected number, based on the Swedish two county study).^5^

None of the screen detected invasive cancers included in the analysis were recurrences. Multiple, multifocal and multicentric cancers occurring at the same time in the same females, were treated as a single event. Five females over the 25 year period, developed a further screen detected primary after being returned to the screening programme, so five females are included as two entries.

The age-standardized invasive cancer detection rate measured by annual SDR with 5 year moving average in shown in Figure 1. The SDR is based on both prevalent and incident screens with expected rates at incident screens adjusted to a 2-year interval. The graph’s marked fluctuations demonstrate the difficulty of monitoring a small screening programme, where small numbers are affected by random fluctuation. In addition, changes to the programme previously described, *e.g.* age changes, increase to 2-yearly, two view screening and alteration in biopsy techniques add further fluctuations. A 5 year moving average is required to correct the annual fluctuations and show the true trend in invasive cancer detection with a marked increase in SDR 1.37 starting around the year 2000. (95% confidence interval for SDR of 1.24–1.50). The distribution of grade of the 400 screen detected invasive cancers is shown in Table 2.

**Table 2.** Distribution of grade of invasive cancers

Grade	Number of Invasive cancers	% of all invasive cancers	% of invasive cancers with known grade
1	102	25.5	31.1
2	170	42.5	51.8
3	56	14.0	17.1
Not Known	72	18.0	-

**Figure 2.**
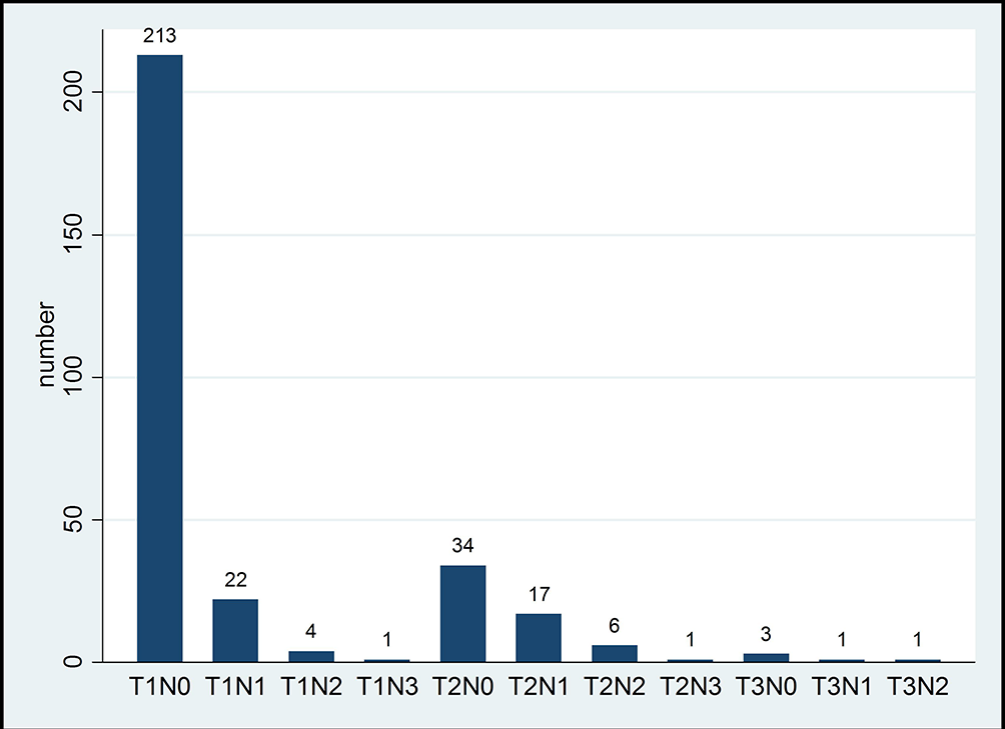
Staging information (where known) for screen detected invasive cancers.

Staging information was recorded for 303 of the 400 screen detected invasive cancers (shown in Table 3 and Figure 2). Of the 303 screen detected invasive cancers 98.3% were Stage I or II showing that the programme is detecting cancers at an early stage (UICC TNM classification).

**Table 3.** Staging information for all invasive cancers detected during screening and as interval cancers

**Stage**	**Screen detected**	**Interval**	**All**
T1N0	213	21	234
T1N1	22	5	27
T1N2	4	1	5
T1N3	1	0	1
T2N0	34	25	59
T2N1	17	7	24
T2N2	6	4	10
T2N3	1	4	5
T3N0	3	1	4
T3N1	1	0	1
T3N2	1	2	3
T3N3	0	1	1
Not Known	97	54	151
**Total**	**400**	**125**	**525**
**Where known**	**Stage I**	**Stage II**	**Stage III** **Stage II+** (*p* < 0.001)
Screen detected Invasive cancer	79.2%	19.14%	1.65% 21%
Interval Cancers	38%	56.3%	5.6% 62%

Staging information for screen detected invasive cancer and interval invasive cancers where available are shown in Table 3. A comparison between the two groups is shown at the bottom of Table 3 as described below. The interval cancers presented with a median of 487 days between screening date as presented in the data with an interquartile range of 364–624 days.

This data are shown graphically in Figure 2.

The missing data for grade and stage is a high percentage, largely reflecting practice in the initial decade, where specialist breast surgeons, pathologists and nurses, attending regular MDT meetings, were not embedded in breast care. Screen detected invasive cancer where available were 79.2% Stage I, 19.14% Stage II and 1.65% Stage III. In contrast, interval cancers were 38% Stage I, 56.3% Stage II and 5.6% Stage III. For screen detected cancers 20.8% (63/303) were Stage II + compared with 62.0% (44/71) of interval cancers (*p* < 0.001). This is seen in Table 3.

The combination of uptake and SDR can be used to estimate percentage mortality reduction^8^ which can be calculated as (uptake/90) x SDR x 31 which gives (85.2/90) x 1.37 × 31=40.2%.

### Objective 2

Classify deaths from invasive breast cancer in females invited for screening in the 25 years, according to presentation (*e.g.* invited but never attended, cancers in lapsed/ceased attenders, interval cancer and screen detected cancer). The total deaths from invasive breast cancer are shown in Table 4.

**Table 4.** Total deaths from invasive breast cancer

**Group**	**Deaths**	**Total in group**	**Risk per 1000** (**95% CI**)
Invited but never attended	40 (29.6%)	2902	13.8 (9.9–18.7)
Attended	95 (70.4%)	1,6866	5.6 (4.6–6.9)
Total	135	19,768	6.8 (5.7–8.1)

CI, confidence interval.

Test of two proportions *p* < 0.001.

The relative risk is given to demonstrate the confounding effect on “invited to screen” statistics. There is a significant but unknown effect from females diagnosed with breast cancer prior to screening invitation in this group. The risk of breast cancer death was therefore 40/2902 (1.38%) compared with 95/16,866 (0.56%) from those who attended screening, a relative risk of 0.41 (*p* < 0.001). Of the 95 who attended screening, 26 (19.22%) were interval cancer; 45 lapsed/ceased (33.7%) and of the 24 screen detected 2 (1.48%) were DCIS and 22 (16.9%) were invasive. Whilst it is not possible to directly attribute deaths prevented to screening attendance these results are used to provide supporting information relating to the effectiveness of the programme and are consistent with the programme aims.

Table 5 shows the classifications of deaths from screening status.

**Table 5.** Classification of deaths from screening status

Total deaths from invasive breast cancer	135
Total deaths from invasive breast cancer invited but did not attend	40 (29.6)
	
The 95 who attended screening:	
Interval cancers	26 (19.22%)
Lapsed/ceased	45 (33.7%)
Screen detected invasive cancers	22
Screen detected DCIS	2
82.2% (111) Breast cancer deaths in invited female not detected at screen	
17.8% (24) Breast cancer deaths in women with screen detected breast cancers.	

DCIS, ductal carcinoma in situ.

### Objective 3

Describe the percentage of females surviving for at least 10 years after primary breast cancer screen detected diagnosis, corrected for deaths from other causes and females lost from the programme (This is an indicative performance measure).

Table 6 shows the screen detected invasive breast cancers in female from 1990 to 31 March 2005, with 10 year follow up to 2015

**Table 6.** 10-year group survival of screen detected invasive cancers

Invasive breast cancers	162
Less exclusions:	
Emigrated	10
Died of other causes	13
Not treated surgically as multiple co-morbidities	1
This leaves 138 cases	
Of these 138 cases, 10-year gruoup survival for screen detected invasive breast cancer	128 (92.8%) (95 % CI = 88.4–97.1%)

Of these 10 emigrated; 13 died of other causes and 1 was not treated surgically because of multiple comorbidities giving a total of 138 females with 10 years follow-up. Of these, 128 (92.8%) had a 10-year group survival for screen detected invasive breast cancer. A more representative 15 year follow up will be assessed in the second quarter of 2020.

Again, whilst such information cannot be definitive it provides useful supporting documentation. The 10-year group survival from screen detected invasive breast cancer must be high if the programme is to reduce mortality, as screening cannot affect breast cancer mortality in all the other groups. These are: (a) Deaths in females invited who do not attend. (b) Deaths in females ceasing/lapsing attendance for screening who develop later cancers. (c) Deaths in females with cancers presenting in the interval between screening mammograms (not detected by screening.). (d) Deaths in females from cancers treated prior to screening, presenting with late metastases.

To counteract this screen unalterable mortality, the programme must detect a large majority of the invasive cancers, at a size and stage where treatment will be effective.

## Discussion

Running a small volume screening programme is a major challenge in terms of monitoring and evaluating the performance of the programme. Small programmes have been linked to poorer performance. Blanks et al in 2002^9^ compared 95 NHS breast screening programme units. The bottom 25% by size were classified as small. These were shown to be performing marginally poorer than medium sized or large programmes, based on individual cancer detection rates. *“The size of the small programmes and the few screen detected cancers (and inherent statistical instability in detection rate) mean that problems are difficult to identify”.^9^*

Giordano et al^4^ surveyed the European screening programmes. 19 programmes performed less than 10,000 tests per screening unit per year with 10 programmes performing less than 5000 screening mammograms per machine per year. They showed 27-fold variation between programmes and state that “This wide variation suggests that programmes with lower unit volumes may require additional efforts and resources to achieve and maintain appropriate quality”.^4^ In a further review in 2015, Giordono et al^10^ states “recall rates and detection rate were better with high volumes of activity compared to lower volumes (those reading <5,000 mammograms per year”.^10^

The anticipated mortality reduction from a screening programme needs to be assessed using, firstly uptake and invasive cancer detection rate and secondly, using interval cancer rates. We have shown that using uptake and SDR the estimated mortality reduction is 40.2% which is a function of the high uptake and good screening performance. The major problem of statistical instability affects interval cancer rates in particular and it may not be possible for a small programme to measure estimated mortality reduction from interval cancers in an appropriate time period as the low annual numbers fail to provide statistical power. Nevertheless, interval cancer rates should be measured and the assessment of false-negative interval cancers is an integral part of any screening quality assurance. Uptake and recall rate are the only two measures that can be adequately assessed using 1 year’s data and as shown it is only with a 5 year moving average that the true invasive cancer detection rate becomes apparent.

Moving 3 or 5 year averages partly compensate but with a small programme of 5000 females screened annually, the annual cancer detection rate shows very large fluctuations which can prevent rapid detection of problems. If geographical or other factors necessitate a small number programme, rigorous quality control of the screening process is vital. In view of this, emphasis needs to be placed on all measures of quality and active participation of screen reading in external assessment programmes such as PERFORMS (Loughborough University) is essential.^11^

The invasive cancers detected at screening need to be predominantly early stage to produce an effect on survival. The invasive breast cancers detected in the Jersey screening programme show a high percentage of females with tumour 20 mm or less (83.1%) and females with negative nodes N0 (83.8%). Based on the Dutch^12^ survival figures, (a 5 year survival rate of 98% for size less than 2 cm and 95–98% for negative node status), the same group of Jersey females (83.1–83.8%) could be predicted to show similar survival. Staging data for screen detected invasive cancers showed 98.3% as Stage I or Stage II. Cancer Research UK^13^ report 5 year survival relative survival 2002–2006 for Stage I females is 99.1%, Stage II females is 87.6% and Stage III females is 55.1%.

Based on the survival data, the programme’s indirect measures demonstrate a likely effect on mortality. Interval cancers showed a significant increase in size, grade and stage relative to screen detected. 26 (20.8%) of the 125 females with interval invasive cancer died compared to 22/400 (5.5%) screen detected invasive cancers and 24/400 (6%) screen detected invasive breast cancer or DCIS.

The mortality percentage figures show 29.6% of deaths from breast cancer occurred in the invited but did not attend group. Accurate reason for failure to attend for screening was not available, but personal observations (PN) show many females had established breast cancer diagnosis before receiving their invitations, so a reasonable assumption would be that failure to attend is frequently because the female is already on a surveillance programme. This group, however, contributes significantly to breast cancer mortality in the invited to screening females. This effect was analyzed by Duffy in 2007^14^ ; “first, it should be noted, that in a given 10 year period, the majority of breast cancer deaths are from tumours diagnosed before that period”. With modern MDT discussion, and effective computers, this reason for females not attending should reduce markedly. Regrettably, in our programme, this effect was not recognized in the first decade of screening, although all new cancers known to the breast unit were identified in the screening programme as “suspended” from 1990 onwards.

The largest contribution to mortality from breast cancer was the group of females lapsing or ceasing screening after reaching the upper age limit. This group contributed 45 (33.3%) of the deaths from breast cancer and further consideration of extending the age limits of screening seems valuable. The results of the age extension trial of the NHS Breast Screening Programme, AgeX,^15^ should provide a significant contribution to this knowledge. Interval cancers contributed 26 (19%) of the deaths from breast cancer meaning that 82% of all the deaths were in females not detected by screening. In contrast, 76% of invasive cancers were detected by screening in the active screening group. Therefore, the reduction in mortality from breast cancer achieved by screening must be produced in the 76% of invasive cancers detected by screening.

To counter bias from lead-time, a 10-year net group survival rate was chosen. This period Jan 1990 to 31 March 2005 encompassed improvements and change in breast cancer treatment. The subsequent 2005–2016 period has shown further improvement in diagnosis and treatment. The 10-year net group survival of 92.8% in females with invasive breast cancer compares with age standardized net 10 year survival rates for UK females 1990–1991 of 60%.^14^ The 92.8% figure is likely to improve with the continuing progress in diagnosis and treatment over the 2005–2016 period.

A 15-year survival rate will be assessed in the second quarter of 2020 which should be less susceptible to lead time bias. Screening has been criticized for detecting early stage indolent non-fatal cancers. Over diagnosis rates, based on academics’ estimates, range from 0 to 54%, implying a lack of firm scientific basis for the estimates. “The methodological approaches differ between studies and there is little agreement in the way the data should be analysed”. After analysis of the selection process and potential biases of estimates, Puliti, Duffy et al^16^ concluded that the most plausible estimates of over diagnosis are in the relatively low range, from 1 to 10% and the higher estimates are likely to be overestimates due to a lack of adjustment for breast cancer risk, and/or for lead time bias. This is reflected in the real world, where autopsy studies of females not known to have breast cancer during life showed invasive breast cancer rates of 0–1.86% (median 1.3%) and DCIS rates of 0–14.7% (median 8.9%).^17^ Based on these studies, giving a maximum invasive breast cancer over diagnosis rate of 2%, 8 out of the 400 invasive cancers in our study could be over diagnosed.

There are a number of strengths and limitations of our study. A small island community enabled a consistent approach to diagnosis and good follow-up information for the screen detected invasive cancers. Accurate information for interval cancers was less available due to some females being treated off-island. Other limitations were partly incomplete registration of females on the database for much of the period of study and the small numbers involved leading to marked fluctuations in cancers detected per year. The 10-year survival times are more accurate than 5 year survival of screen detected cancers as this allows partial correction for lead time bias, but a stronger period of 15 year survival would be preferable when numbers become available.

## Conclusion

A small volume screening programme can provide an effective level of screening. The high percentage of females with tumour size of less than 20 mm and negative nodes is likely to lead to increased survival and the combination of uptake and SDR can be used to provide an estimate of the likely mortality reduction that will be achieved. Above all, for a small screening programme quality assurance can provide great confidence that the programme is functioning effectively.

As the uptake is greater than the UK mainland and closer to the uptake in some of the Swedish Trials the predicted mortality benefit at 40% is higher. Even although based on small numbers the results are consistent with expectations and provide support to the suggestion that breast cancer screening is having a major impact on reducing breast cancer mortality. The results from Jersey suggest a small breast screening programme can maintain national screening quality and that quality assurance is essential especially where numbers on an annual basis are small.

Even with just uptake and SDR evaluated over longer time periods it is possible to provide confidence that the programme is performing well. It would be tempting to think that because of small numbers being reported annually that QA is not a worthwhile process, but the experience of Jersey shows that this is not the case. The results provided demonstrate that a screening programme with marginal annual numbers, compared to UK recommendations, can achieve a high standard of screening, at a level predicted to produce a reduction in mortality from breast cancer.
