# Supplementary figures and images for: Effectiveness of a small breast screening programme: 25 year evaluation (25 year breast screening evaluation)
Source: BJR Open. 2019 Jul 13;1(1):20180018. doi: 10.1259/bjro.20180018 (PMC7592412; doi:10.1259/bjro.20180018)

Appendix I:

Current National Minimum Standards for the NHSBSP (16)


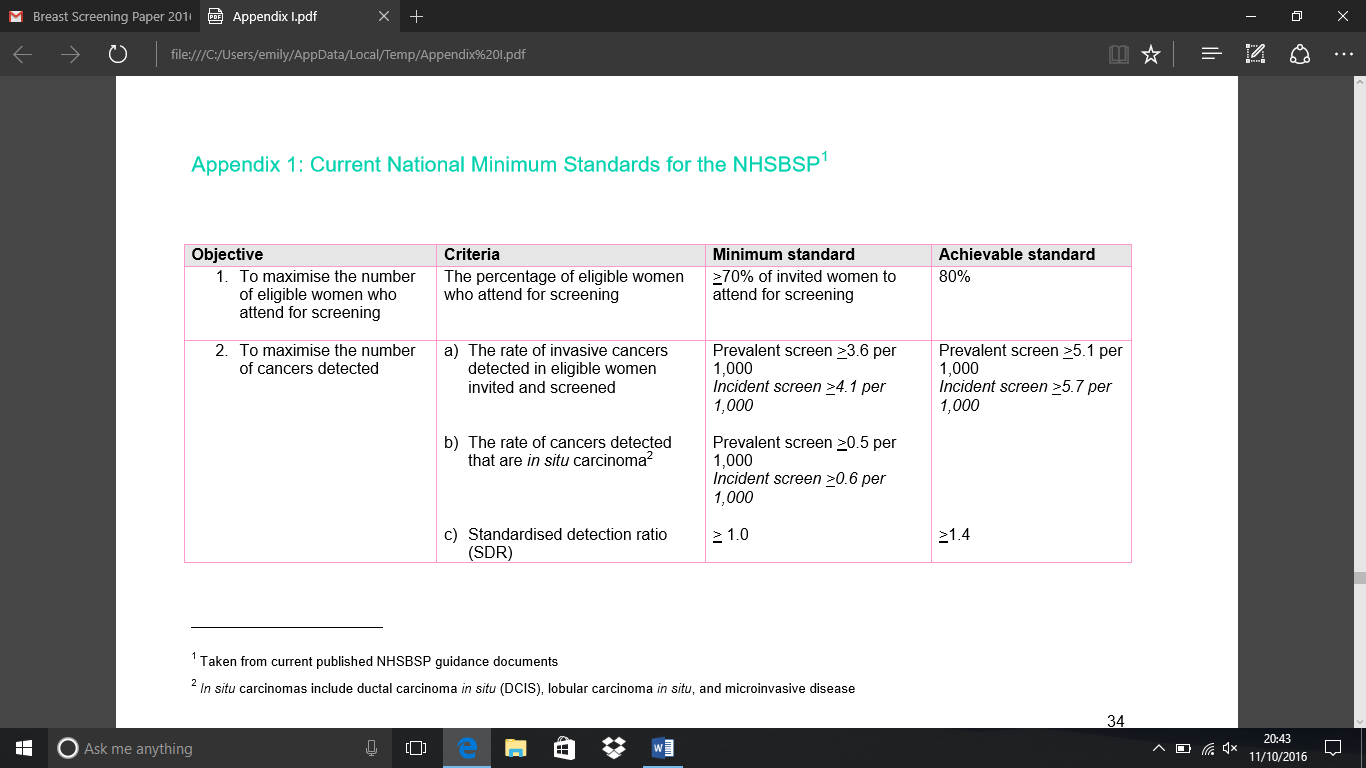


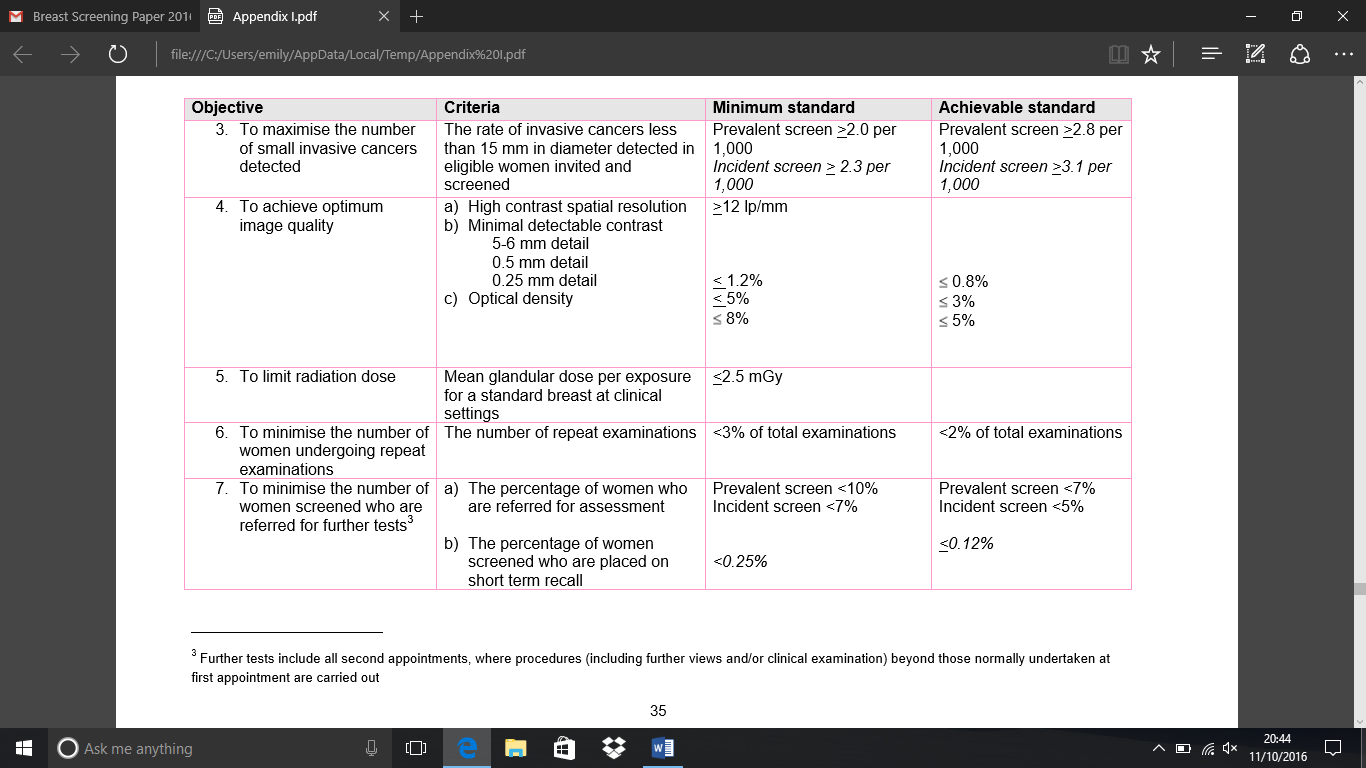


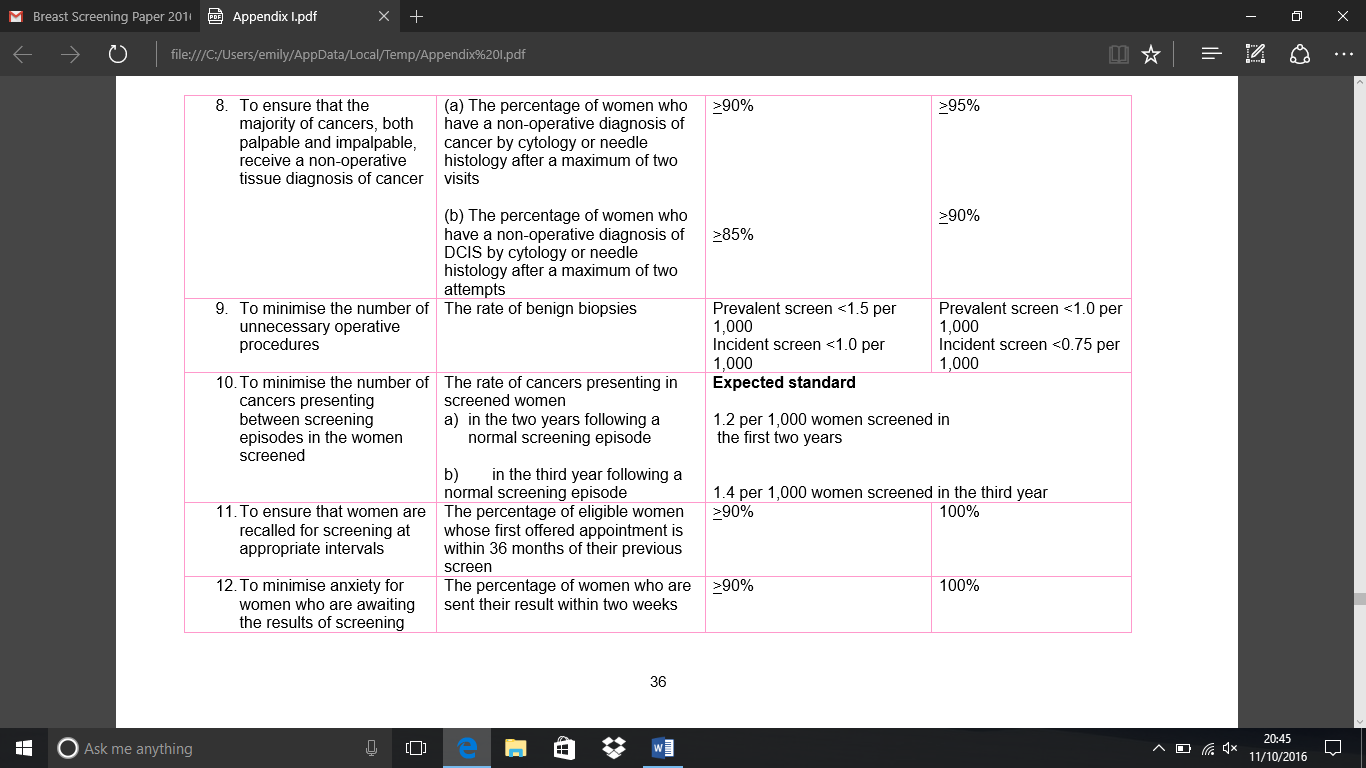

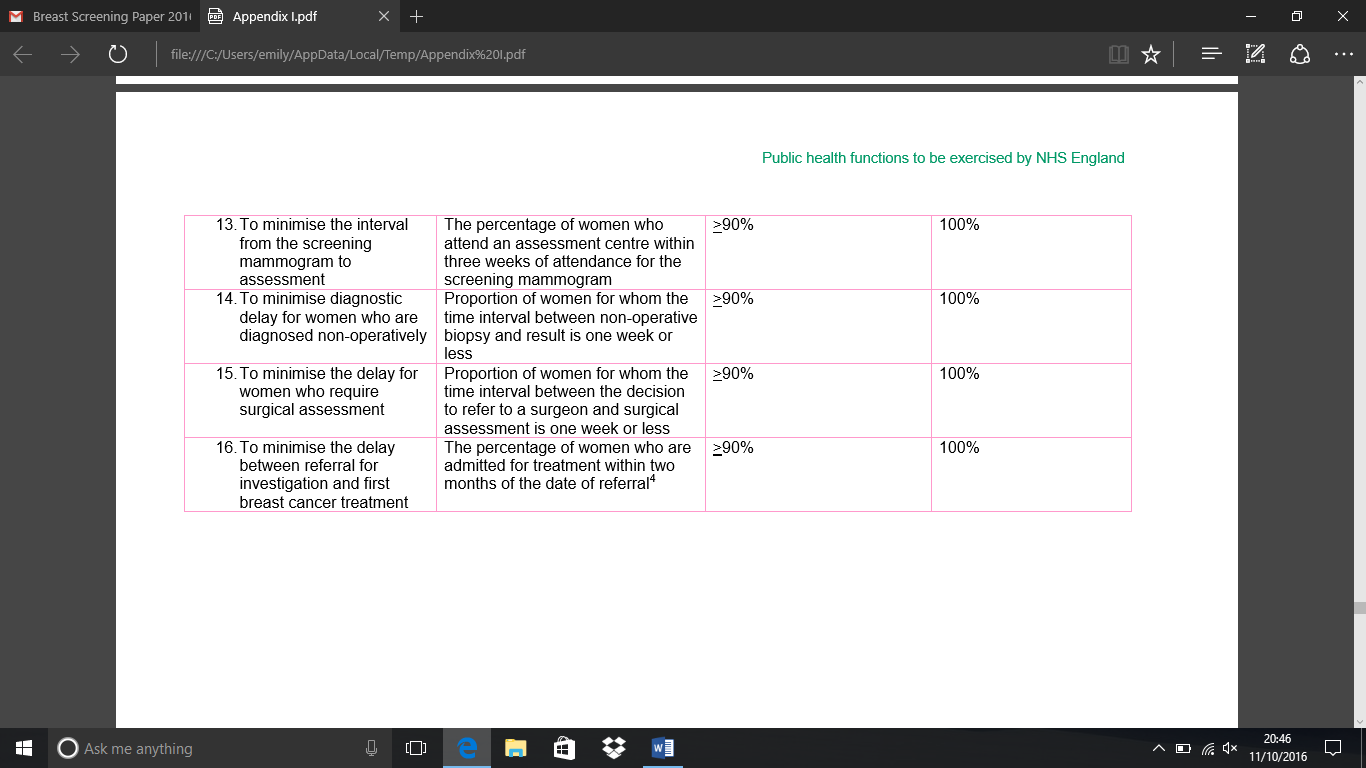

Supplement: Supplemental Material [file bjro.20180018.suppl-01.docx]
